# Novel function of MDA-9/Syntenin (SDCBP) as a regulator of survival and stemness in glioma stem cells

**DOI:** 10.18632/oncotarget.10851

**Published:** 2016-07-26

**Authors:** Sarmistha Talukdar, Swadesh K. Das, Anjan K. Pradhan, Luni Emdad, Xue-Ning Shen, Jolene J. Windle, Devanand Sarkar, Paul B. Fisher

**Affiliations:** ^1^ Department of Human and Molecular Genetics, Virginia Commonwealth University, School of Medicine, Richmond, VA, USA; ^2^ VCU Institute of Molecular Medicine, Virginia Commonwealth University, Richmond, VA, USA; ^3^ VCU Massey Cancer Center, Virginia Commonwealth University, Richmond, VA, USA

**Keywords:** MDA-9/Syntenin (SDCBP), stemness, glioma stem cells, survival, apoptosis

## Abstract

Glioblastoma multiforme (GBM) is an aggressive cancer with current therapies only marginally impacting on patient survival. Glioma stem cells (GSCs), a subpopulation of highly tumorigenic cells, are considered major contributors to glioma progression and play seminal roles in therapy resistance, immune evasion and increased invasion. Despite clinical relevance, effective/selective therapeutic targeting strategies for GSCs do not exist, potentially due to the lack of a definitive understanding of key regulators of GSCs. Consequently, there is a pressing need to identify therapeutic targets and novel options to effectively target this therapy-resistant cell population. The precise roles of GSCs in governing GBM development, progression and prognosis are under intense scrutiny, but key upstream regulatory genes remain speculative. MDA-9/Syntenin (SDCBP), a scaffold protein, regulates tumor pathogenesis in multiple cancers. Highly aggressive cancers like GBM express elevated levels of MDA-9 and contain increased populations of GSCs. We now uncover a unique function of MDA-9 as a facilitator and determinant of glioma stemness and survival. Mechanistically, MDA-9 regulates multiple stemness genes (*Nanog, Oct4 and Sox2*) through activation of STAT3. MDA-9 controls survival of GSCs by activating the NOTCH1 pathway through phospho-Src and DLL1. Once activated, cleaved NOTCH1 regulates C-Myc expression through RBPJK, thereby facilitating GSC growth and proliferation. Knockdown of MDA-9 affects the NOTCH1/C-Myc and p-STAT3/Nanog pathways causing a loss of stemness and initiation of apoptosis in GSCs. Our data uncover a previously unidentified relationship between MDA-9 and GSCs, reinforcing relevance of this gene as a potential therapeutic target in GBM.

## INTRODUCTION

Cancer is multifactorial in its etiology and multistep in its evolution [1]. Glioblastoma multiforme (GBM), the most common form of, is an aggressive cancer that causes high mortality and morbidity. GBM currently remains one of the most difficult cancers to treat, with less than a 5% 5-yr survival rate, despite multi-modality therapies including surgery, radiation therapy, and chemotherapy [2]. This is potentially due to a lack of well-defined understanding of the mechanism(s) underlying GBM's complex heterogeneity, plasticity and therapy resistance. Since their discovery and initial characterization in 1994, research on cancer stem cells has intensified, providing convincing evidence that these unique cells are major contributors to cancer growth, progression, tumor heterogeneity and resistance to therapeutic intervention [3]. The concept that cancer is comprised of nearly homogenous, ectopically growing cells has been replaced with a more complex heterogeneous model in which cancer cells have varied potential to metastasize, interact with the stroma and regrow after therapy (relapse) [3–6]. Gliomas are organized as cellular hierarchies that are generated from and maintained by subpopulations of self-renewing glioma stem cells (GSCs) [6–9]. These subpopulations of cancer stem cells display high tumorigenic potential and stemness properties and have been isolated from cancer patients with various types of tumors [9]. The current consensus is that tumors comprised of cells with stem-like characteristics portend a poorer prognosis, which have important clinical implications for cancer diagnosis and treatment [6–12]. Presence of a high proportion of GSCs also permits stratification of patients into a high metastatic risk group and represents an important area of clinical investigation [8].

Isolation of stem cells from different normal and cancerous tissues has been facilitated by the identification of specific cell surface markers. Recently, two mutually exclusive GSC subtypes: pro-neural and mesenchymal, were identified and characterized with distinct dysregulated signaling pathways [13]. CD133/ Prominin-1 is an established and broadly accepted pro-neural GBM stem cell marker [9, 13, 14] that is also shared with other cancer stem cells from melanoma, prostate, pancreatic, liver, colon, lung, and ovarian cancers [3]. Recently, the importance of CD44 has been recognized as a marker of mesenchymal GBM stem cells [13], as well as prostate and breast cancer stem cells [3, 11].

In addition to cell surface markers, several pathways and molecules that control self-renewal and differentiation of cancer stem cells and normal stem cells have been identified including p-STAT3, NOTCH, C-Myc, NANOG, OCT4, SOX2 and others [3, 15, 16]. These regulators of stemness also influence tumorigenesis and tumor progression [17]. NOTCH and STAT3 signaling play critical roles in stem cell fate determination. OCT4, SOX2, and NANOG are central transcriptional regulators of stemness, establishing an interconnected autoregulatory network to sustain cell pluripotency and self-renewal [15]. NOTCH1, SOX2, and CD133 are known to regulate the pro-neural GSC subtype, whereas CD44 is believed to regulate the mesenchymal GSC subtype [13]. Moreover, many aggressive cancers that result in poor patient survival show higher expression of these stemness genes [18, 19]. Despite clinical significance, effective/selective targeting strategies for cancer stem cells, including GSCs, do not currently exist [20].

MDA-9/Syntenin (SDCBP) is a scaffold protein that interacts with a remarkable repertoire of key regulatory proteins, including SRC, FAK and EGFR, which are often related to expression of the tumor phenotype and cancer progression [21, 22]. MDA-9 is a diagnostic marker of tumor aggression and grade in gliomas [23], melanomas [24, 25], and breast cancer [26]. Based on these observations, we hypothesized that higher tumor grade, which correlates with a more invasive and metastatic phenotype, might consist of an increased proportion of GSCs that would express elevated levels of MDA-9. GSCs are major contributors to cancer progression [9] and MDA-9 plays a seminal role in the progression of several cancer types including glioma [21–26]. Accordingly, we currently assessed the association between stemness and MDA-9 expression in GBM, as well as in normal astrocytes. Stemness is defined as the ability of stem cells to self-renew and differentiate [27]. We studied this property using sphere formation assays, cell-surface based stem population assessment, monitoring genes regulating self-renewal, and tumorigenicity. Additionally, the influence of MDA-9 on GSC survival, growth, angiogenesis and chemoresistance was also examined. Finally, we dissected the mechanisms contributing to MDA-9-mediated stem phenotypes and survival. Our studies now demonstrate for the first time that MDA-9 promotes glioma stem cell phenotypes and survival through regulation of NOTCH1, C-Myc, STAT3 and *Nanog* in GSCs.

## RESULTS

### *mda-9* regulates stemness in normal astrocytes and glioma cells

**Figure 1:**
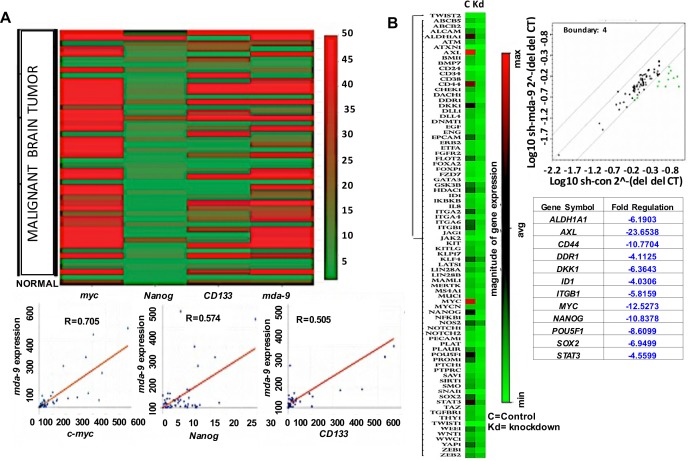
*mda-9* expression correlates with stemness markers in clinical samples A Clinical array data confirms a strong correlation between expression of *mda*-9 with the stemness markers *c-myc*, *Nanog* and *CD133*. **B.** GSC array data demonstrates dramatic downregulation of stem cell markers in *mda*-9 knockdown (kd) GSCs. See also Figure S1.

**Figure 2:**
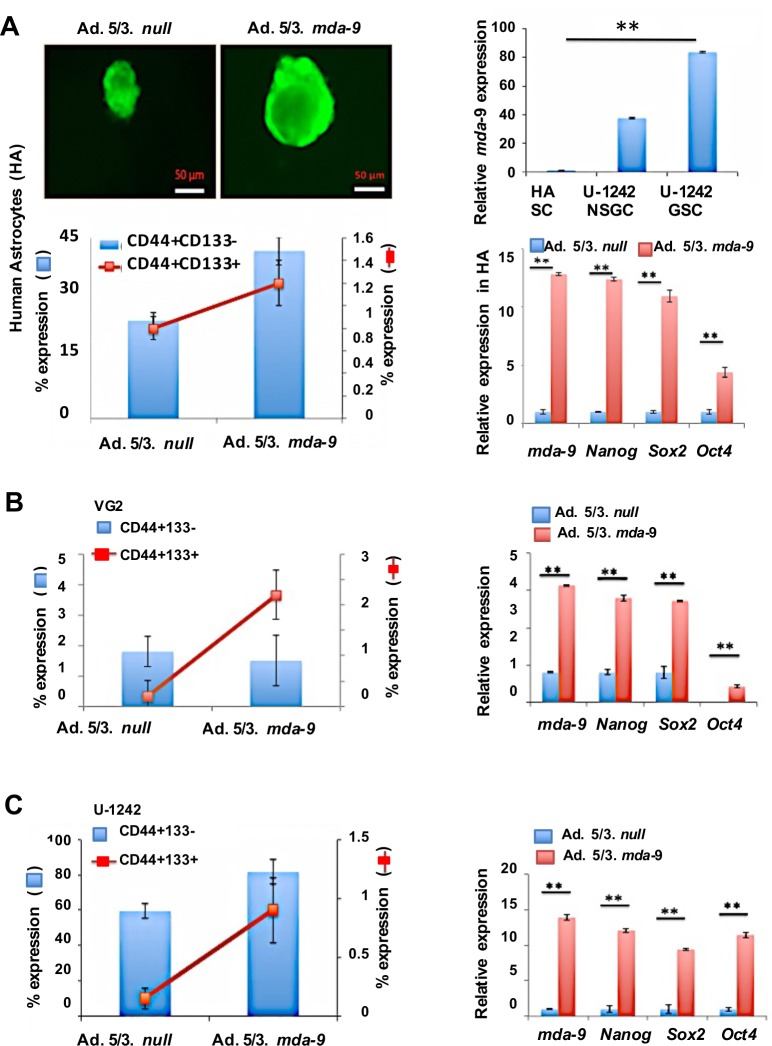
Overexpression of *mda-9* enhances stemness markers in normal astrocyte stem cells and GSCs **A.** Left upper panel, live image analysis of human primary astrocyte (HA) stem cell neurospheres. Left lower panel, FACS analysis of stem cell (SC) markers in null vector- and *mda-9*-overexpressing HA neurospheres. Right upper panel, *mda-*9 expression in HA stem cells as compared to U-1242 NSGCs (non stem glioma cells) and GSCs (stem cells). Right lower panel, overexpression of *mda*-9 significantly enhances several stem cell markers in HA cells. **B.** FACS (left) and RT-PCR (right) analysis of stem cell (SC) markers and stemness genes in null vector and *mda-9*-overexpressing VG2 non stem glioma cells. **C.** FACS (left) and RT-PCR (right) analysis of stem cell (SC) markers and stemness genes in null vector and *mda-9*-overexpressing U-1242 non stem glioma cells. Relative expression indicates fold-change in expression. Bars represent SEM. *P < 0.05, **P < 0.01 using student *t*-test and ANOVA. SCs = stem cells. See also Figure S2, Table 1, and 2.

A positive correlation between *mda-9* expression and stemness was evident in GBM (Figure 1A, 1B and Figure S1A). Forty-eight patient samples were assayed for *c-myc*, *CD133*, *Nanog* and *mda-9* expression (Figure 1A, Figure S1A). Data was normalized to 18S and beta tubulin expression and analyzed statistically by multiple regression analysis. The results were statistically significant (R^2^ = 0.743, *p* < 0.05), and a positive correlation was observed between *mda-9* and *myc* (R = 0.705), *Nanog* (R = 0.574) and *CD133* (R = 0.505) expression (Figure 1A). Considering these observations, we assayed control and *mda-9* knockdown (kd) (sh*mda-9*) GSCs from a clinical GBM sample (VG2) using a cancer stem cell array (Human Cancer Stem Cells RT^2^ Profiler PCR array, Qiagen/Sabiosciences) (Figure 1B). Eighty-four genes were examined, and kd of *mda-9* significantly affected a spectrum of pluripotency genes and the STAT3 pathway. The genes most affected by *mda-9* kd in GSCs (downregulated a minimum of ~4-fold by selecting the statistical boundary for Log_10_sh*mda-9* del del CT/ Log_10_shcon del del CT as 4) were *ALDH1A1, AXL, CD44, DDR1, DKK1, ID1, ITGB1, MYC, NANOG, OCT4/POU5F1, SOX2* and *STAT3* (Figure 1B). All of these genes, except for DKK1, promote stemness. Additionally, *AXL* is an important target for chemoresistance [28]. An increase in *mda-9* expression was also evident in GSCs > non-stem glioma cells (NSGCs) > normal stem cells (SCs) (Figure 2A).

*mda-9* mRNA levels were quantified in different stem and non-stem cell populations of gliomas, from both cell lines and clinical samples. In all samples, increased *mda-9* expression was observed in stem *vs*. non-stem populations (Table 1). *mda-9* expression in non-stem U-1242 cells, NSGCs, was ~35-fold greater than in primary adult human astrocyte (HA) SCs (Figure 2A, top right panel). Additionally, the expression of *mda-9* in U-1242 GSCs was double that of U-1242 NSGCs (Figure 2A, top right panel). Since GSCs expressed higher levels of stemness genes than corresponding non-stem cells, we examined the relationship between *mda-9* expression and stemness in GSCs *vs*. NSGCs. Elevated *mda-9* expression directly correlated with stemness (Table 1), i.e., *mda-9:Nanog* (Pearson's correlation coefficient R = 0.838, coefficient of determination R^2^ = 0.7034), *mda-9:Sox2* (R = 0.968, R^2^ = 0.937), *mda-9:Oct4* (R = 0.836, R^2^ = 0.698) and *mda-9:c-myc* (R = 0.954, R^2^ = 0.911).

**Table 1:** Expression of *mda-9* and stemness genes in non-stem glioma cells (NSGCs) and glioma stem cells (GSCs)

CELL LINE/SAMPLE	VG2		VG9		U-1242	
GENES	Non-stem glioma cell	Glioma stem cell	Non-stem glioma cell	Glioma stem cell	Non-stem glioma cell	Glioma stem cell
*mda-9*	1±0.04	6.7±1.20	1±0.20	5.2±0.44	1±0.03	10.4±0.12
Stemness *Nanog Sox2 Oct4 c-myc*						
	1±0.20	15.7±0.46	1±0.05	11.5±0.79	1±0.07	11.2±2.20
	1±0.07	2.0±0.70	1±0.03	2.0±0.82	1±0.48	1.8±0.08
	1±0.09	19.8±2.70	1±0.31	15.6±1.54	1±0.90	5.5±0.25
	1±0.42	9.1±0.81	1±0.02	8.7±0.05	1±0.10	10.3±1.03
*Notch1*	1±0.06	4.1±0.15	1±0.10	3.5±0.03	1±0.61	3.7±0.19

Forced *mda-9* overexpression in normal human astrocytes led to a significant increase in spheroid size (Figure 2A, top left panel), stem populations (Figure 2A bottom left panel), self-renewal and pluripotency (Figure 2A bottom right panel, Figure S1B) as reflected by assessment of putative GSC and NSGC populations as well as changes in genes involved in self-renewal. No change in tumorigenicity was observed, when assayed by mice xenograft studies (data not shown). Overexpression of MDA-9 in NSGCs, significantly increased the stem population and expression of canonical stem regulatory genes (Figure 2B, 2C). Even though NSGC populations had elevated expression of *mda-9*, the GSC populations had significantly higher expression than the corresponding normal brain (Figure 2A, top right panel). To further confirm that MDA-9 regulates stem regulatory genes *mda-9* was suppressed by kd in GBM (cell line and clinical samples). Silencing of *mda-9* significantly decreased the recognized stem regulatory genes, and markers (Table 2). Overall, *Nanog* was decreased by ~33-, ~25- and ~11-fold, *Oct4* by ~7-, ~12- and ~2-fold, and *Sox2* by ~10-, ~7- and ~4-fold in the *mda-9* kd GSCs from VG2, VG9, and U-1242, respectively. Silencing of *mda-9* also resulted in significant loss of self-renewal (Figure S1B) as defined by the self-renewal assays. In total, these data support the hypothesis that *mda-9* can regulate stemness in both normal astrocyte stem cells and GSCs.

**Table 2:** Expression of *mda-9* and stemness genes in control and sh*mda-9* GBM GSCs

GENES	U-1242		VG2		VG9	
	sh*con*	sh*mda-9*	sh*con*	sh*mda-9*	sh*con*	sh**mda-9**
*mda-9*	1±0.20	0.10±0.01	1±0.02	0.10±0.01	1±0.36	0.12±0.01
Stemness genes *Nanog Sox2 Oct4 c-myc*						
	1±0.19	0.09±0.01	1±0.04	0.03±0.10	1±0.42	0.04±0.02
	1±0.11	0.22±0.06	1±0.53	0.10±0.03	1±0.53	0.15±0.05
	1±0.03	0.45±0.03	1±0.34	0.15±0.03	1±0.30	0.08±0.02
	1±0.41	0.11±0.02	1±0.19	0.09±0.02	1±0.25	0.06±0.01

### *mda-9* influences self-renewal through STAT3

**Figure 3:**
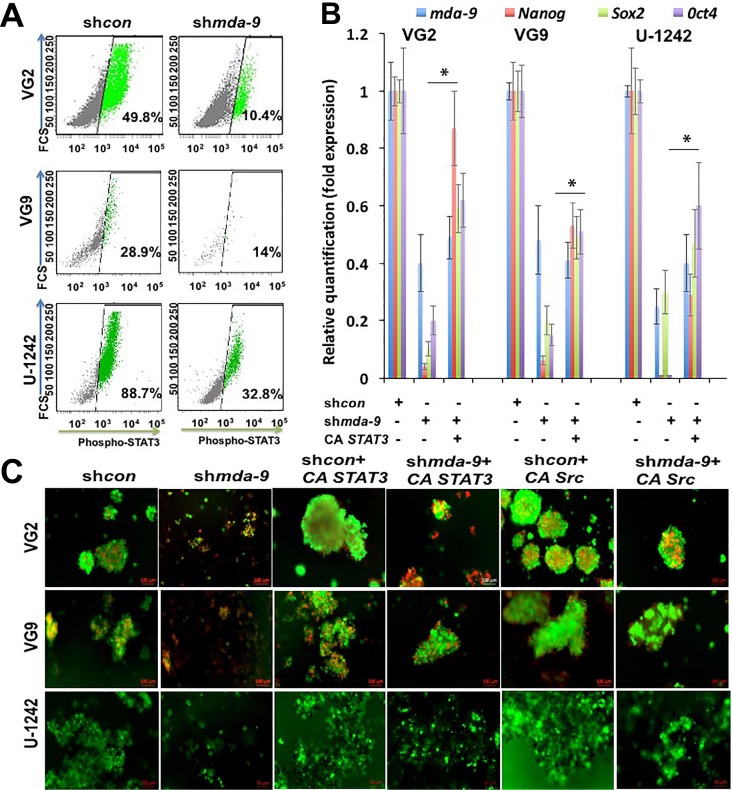
STAT3 activation is downstream of *mda-9* **A.** Flow cytometry analysis of p-STAT3 in control and *mda-9* kd GSCs from clinical GBM (VG2, VG9) and the GBM cell line U-1242. **B.** RT-PCR analysis for expression of *mda-9* and stemness genes in sh*con*, *mda-9* kd, and *mda-9* kd cells overexpressing constitutively active (CA) *STAT3*. Relative expression indicates fold change in expression. **C.** Image analysis of sh*con*, *mda-9* kd (sh*mda-9*) and sh*con* or *mda-9* kd cells overexpressing constitutively active (CA) *STAT3* or CA *Src*; live cells are green, dead cells are red, *P < 0.05, using student *t*-test and ANOVA. * indicates significance between sh*mda-9* and sh*mda-9 +* CA *STAT3* groups. See also Figure S2 and Figure S3.

**Figure 4:**
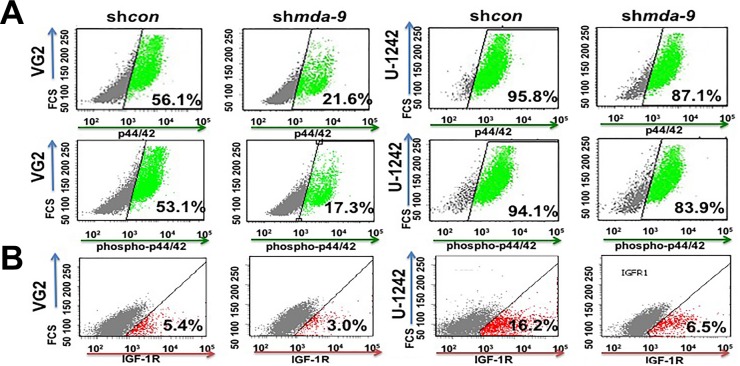
*mda-9* indirectly regulates STAT3 activity **A.** Flow cytometry analysis of p44/42, phosphor-p44/42 and **B.** IGF-1R in sh*con* and *mda-9* kd GSCs from GBM clinical samples, and cell line. See also Figure S2.

STAT3 is indispensable for the regulation of self-renewal in human stem cells including GSCs [18, 29, 30]. Considering this seminal role of STAT3, we investigated the effect of *mda-9* expression on STAT3. Kd of *mda-9* significantly decreased the expression of p-STAT3 (Figure 3A; Figure S2). p-STAT3 expression was decreased ~2-4-fold overall in sh*mda-9* cells (32.0 ± 6.3% decrease in VG2; 12.1 ± 3.9% decrease in VG9; 40.0 ± 6.0% decrease in U-1242). To further confirm our hypothesis, we overexpressed *mda-9* in primary human astrocytes and found that *mda-9* overexpression led to a significant increase in p-STAT3 (Figure S2). The effects of *mda-9* silencing were significantly attenuated by overexpressing a constitutively active STAT3 (A662C/N664C; *CA STAT3*) (Figure 3B and 3C). Since active SRC positively regulates STAT3 [31], we overexpressed constitutively active SRC (Y529F; *CA Src*) and once again observed significant recovery of *STAT3* function in the sh*mda-9* cells (Figure 3C). However, overexpression of a non constitutively-active SRC (NCA *Src*) failed to enhance *STAT3* rescue function in the sh*mda-9* GSCs (Figure S3). Since STAT3 is also regulated by p44/42 and IGF-1R [32, 33], we also measured the expression of these proteins in control and sh*mda-9* GSCs. We observed some decrease in p44/42, a significant decrease in phospho-p44/42 (31.4 ± 6.2% decrease in VG2; 62.0 ± 7.9% decrease in VG9; 9.5 ± 2.7% decrease in U-1242) (Figure 4A; Figure S2), and IGF-1R in the sh*mda-9* cells (Figure 4B, ~2 and ~3-fold in VG2 and U-1242, respectively).

### MDA-9 regulates stem cell survival and intracranial GSC growth

**Figure 5:**
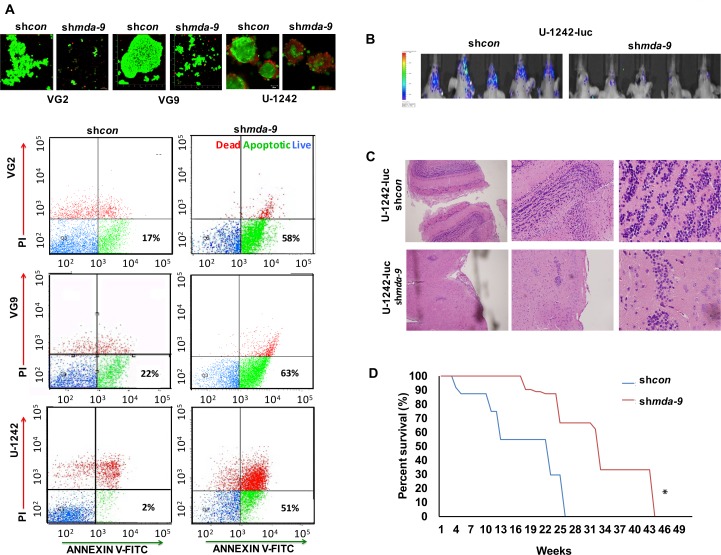
Suppression of *mda-9* expression decreases GSC viability, tumorigenesis and enhances mouse survival **A.** upper panel: Live/dead fluorescent images in GBM clinical samples (VG2, VG9; live cells are green, dead cells are red) and cell line (U-1242), lower panel: flow cytometry analyses in GSCs show increased percentage of cell death and apoptosis caused by kd of *mda-9*. **B.** Bioluminescent imaging (BLI) of intracranial GBM, shows intense luciferase activities in mice with sh*con* U-1242-Luc as compared to the *mda-9* kd U-1242-Luc group (*n* = 10). **C.** Images of H&E staining of tissue collected from sh*con* and sh*mda-9* intracranial orthotopic brain tumors at 40, 100, and 400x magnification. **D.** Survival analysis of mice plotted over time showing the cumulative effect of *mda-9* kd in GSCs. Knocking down *mda-9* increased survival time (*p* = 0.04, log rank test) relative to control. *p < 0.05.

*mda-9* kd increased apoptotic cell death in GSCs (Figure 5A). The population of apoptotic cells in sh*mda-9* GSCs was 38 ± 3.3 %, 36 ± 5.1 % and 45 ± 4.9 % (in VG2, VG9 and U-1242, respectively) after 72 hours, which was ~5-fold of that observed in sh*con* GSCs. Suppression of *mda-9* also resulted in a significant loss in GSC tumorigenicity and an increase in mouse survival *in vivo* (Figure 5B, 5C, 5D; *p* < 0.05). The control mice showed spongioblastic tumors with rhythmic palisades, a constant feature of aggressive high grade glioblastoma. Tumors in mice injected with sh*mda-9* GSCs were extremely small, and did not display the distinguishing aggressive spongioblastic pattern (Figure 5C).

### MDA-9 regulates GSC survival through NOTCH1 signaling

**Figure 6:**
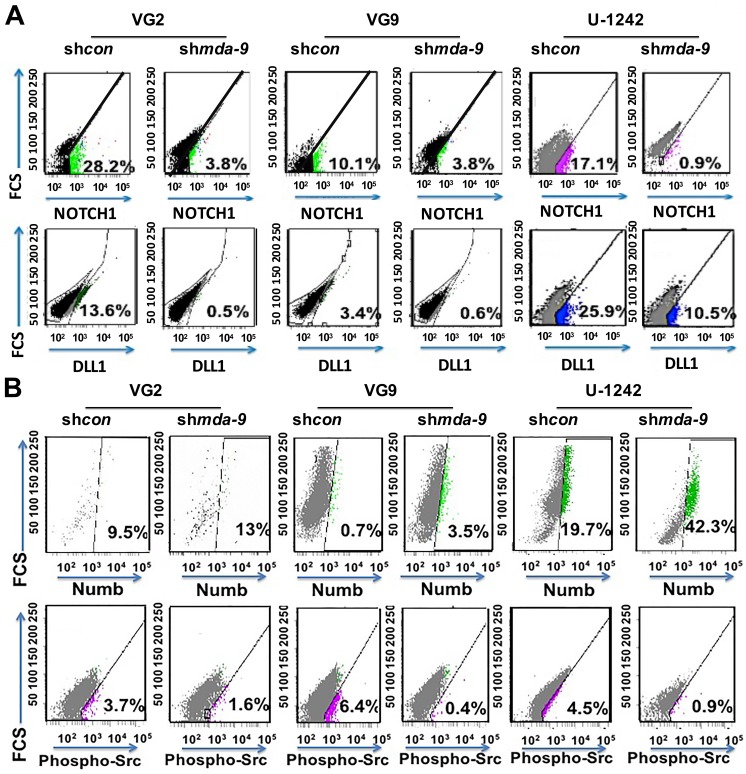
*mda-9* regulates the NOTCH1 pathway by regulating NOTCH1 degradation and activation **A.** Flow cytometry analyses of control and *mda-9* kd GSCs from GBM clinical samples (VG2 and VG9) and cell line (U-1242) for surface expression of NOTCH1 and DLL1. **B.** Flow cytometry analyses of control and *mda-9* KD GSCs from GBM clinical samples and cell lines for NUMB and p-SRC expression. See also Figure S2.

**Figure 7:**
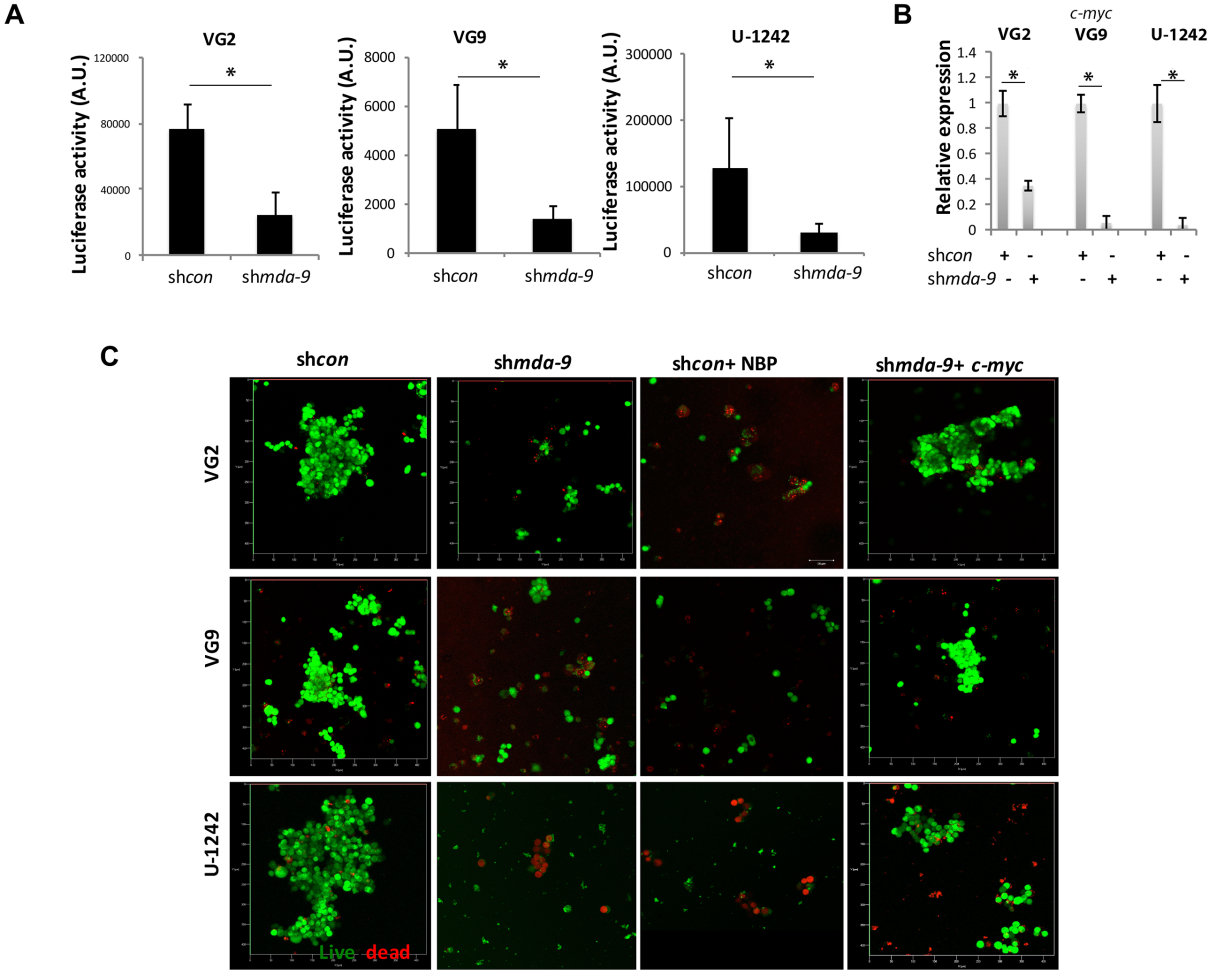
*mda-9* regulates GSC survival by controlling c-*myc* through the NOTCH1/RBPJK pathway **A.** Luciferase reporter assay analysis of control and *mda-9* kd GSCs from GBM clinical samples (VG2 and VG9) and cell line (U-1242) for RBPJK promoter activity. **B.** RT-PCR-based *c-myc* expression in control and *mda-9* kd GSCs from GBM cells. Relative expression indicates fold-change in expression. **C.** Peptide blocking and recovery of function studies to elucidate the effect of Notch1 blocking peptide (NBP) and *c-myc* overexpression on sh*con* and *mda-9* kd GBM GSCs, respectively; live cells are green, dead cells are red. Bars represent SEM. *P< 0.05, **P < 0.01 using student *t*-test and ANOVA. See also Figure S2.

NOTCH1 expression was decreased 6.9 ± 0.5, 2.1 ± 0.6, and 16.5 ± 2.3-fold following kd of *mda-9* in VG2, VG9 and U-1242 GSCs, respectively (Figure 6A; Figure S2). Decreased *mda-9* expression led to NOTCH1 degradation through increased expression of NUMB (1.3 ± 0.7, 4.8 ± 0.4, 2 ± 0.5-fold increase, respectively) and decreased p-SRC expression (2 ± 0.9, 15.8 ± 1.2, 5.5 ± 0.4-fold decrease in relative expression, respectively) in VG2, VG9, and U-1242 GSCs (Figure 6B). *mda-9* kd also caused a loss of NOTCH1 activation (~7-fold in VG2, ~2.6-fold in VG9, and ~19-fold in U1242) as well as ~27- (VG2), ~6- (VG9) and ~2- (U-1242) fold reduction of DLL1 (Figure 6A). Blocking NOTCH1 recapitulated the phenotype observed with *mda-9* kd (Figure 7C). The decreased activity of NOTCH1 in sh*mda-9* cells lead to a significant decrease in RBPJK (Immunoglobulin Kappa J Region Recombination Signal Binding Protein 1) expression (Figure 7A). The effect of *mda-9* kd was rescued by expressing a constitutively active SRC (CA *Src*), but not with a non constitutively-active SRC (NCA *Src*). (Figure S3). Additionally, partial recovery from *mda-9* kd occurred with addition of a DLL1 peptide (Data not shown).

### MDA-9 regulates stem cell renewal, maintenance and survival through C-Myc

Considering C-Myc's influential role in stem cell renewal, maintenance, and survival [34, 35], we investigated the role of MDA-9-mediated regulation of C-myc in HA SCs and GSCs. Suppression of *mda-9* by kd and enhanced expression of *mda-9* with an expression vector lead to a significant decrease (~3-, ~2- and ~5-fold protein, and ~3-, ~10- and ~12-fold mRNA in VG2, VG9 and U-1242 GSCs, respectively) or g*ain* of C-Myc (~3-fold protein in HA) expression, respectively (Figure 7B; Figure S2; Table 2). The change in C-Myc was observed at both an RNA and protein level (Figure 7B; Figure S2; Table 2). This loss of a *c-myc* expression phenotype in sh*mda-9* GSCs was reversed by *c-myc* overexpression (Figure 7C). *mda-9* regulation of *c-myc* occurred though RBPJK transcription (Figure 7A); which is possibly regulated by NOTCH1 cleavage/activation (Figure S2) *via* interaction with its ligand, DLL1 (Figure 6A). These findings support the concept that MDA-9 plays a critical role in the regulation of C-Myc in GSCs, which is a major contributor of glioma stemness and GSC survival (34) *via* the activation of NOTCH1 and RBPJK.

### MDA-9 regulates GSC survival through p27/Kip-1 and cIAP2

**Figure 8:**
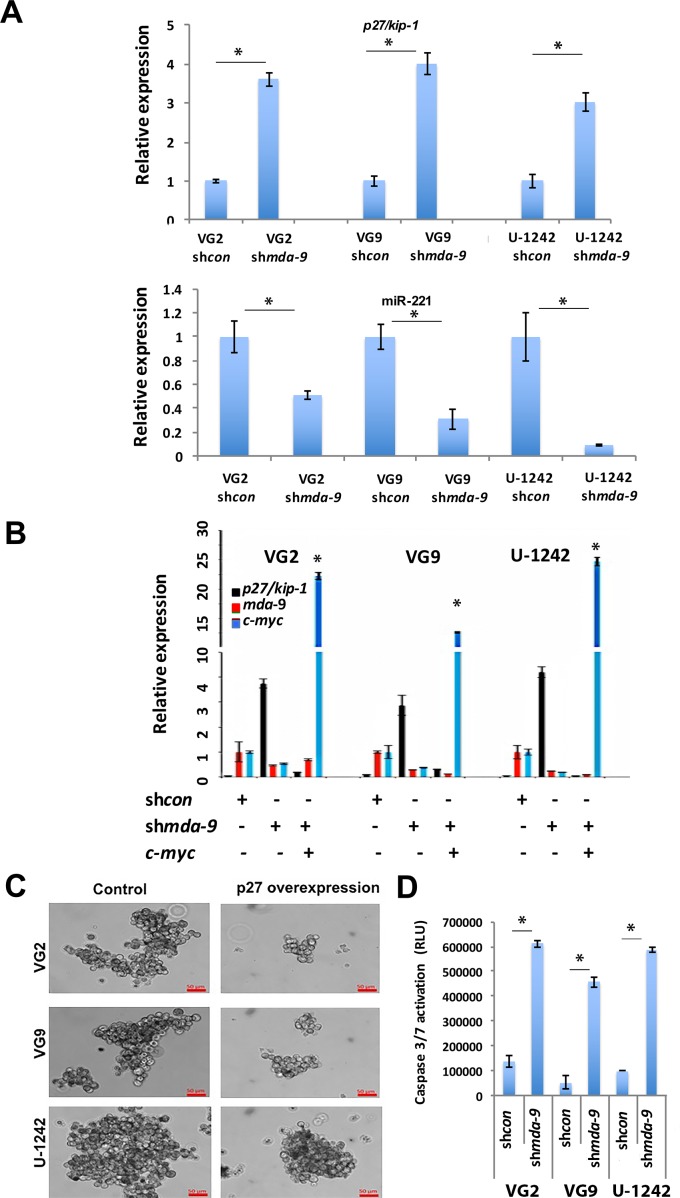
*mda-9* regulates apoptosis by p27/Kip-1 expression through the NOTCH1/*RBPJK/C*-Myc pathway **A.** RT-PCR analysis of *p27/Kip-1* and miR-221 expression in sh*con* and *mda-9* kd GBM GSCs. **B.** RT-PCR analysis of *p27/kip-1*, *mda-9*, and *c-myc* in sh*con*, *mda-9* kd GSCs and *mda-9* kd GSCs overexpressing *c-myc.* * indicates significance in expression of *p27/kip-1* and *c-myc* between the sh*mda-9* and sh*mda-9* + *c-myc* groups. Relative expression indicates fold-change in expression. See also Figure S2. **C.** Image analysis of control and p27/Kip-1 overexpressing GSCs. **D.** Caspase 3/7 activation analysis in sh*con* and sh*mda-9* GSCs. Bars represent SEM. *P < 0.05, using student *t*-test and ANOVA.

Kd of *mda-9* led to increased expression of p27/Kip-1 in GSCs, at both an RNA and protein level (Figure 8A, Figure S2). The increased expression of p27 that culminated in cell death could be prevented by forced expression of *c-myc*, indicating that GSC survival is dependent on *c-myc* and *p27/kip-1* expression (Figure 8B). In sh*mda-9* GSCs, expression of miR-221 was also significantly decreased (Figure 8A). These findings demonstrate that *p27/kip-1* is regulated by *mda-9* through *c-myc* and miR-221. *mda-9* kd caused decreased *cIAP2* expression (Figure S2) and this combined with increased expression of *p27/kip-1* in sh*mda-9* GSCs may amplify GSC death. To verify *p27/kip-1*'s involvement in GSC survival, we overexpressed *p27/kip-1* in GSCs and observed a loss of sphere integrity and viability, in both patient-derived GBM and the U-1242 GBM cell line (Figure 8C). We also observed that cell death in sh*mda-9* GSCs was mediated by Caspase activation (Figure 8D).

## DISCUSSION

**Figure 9:**
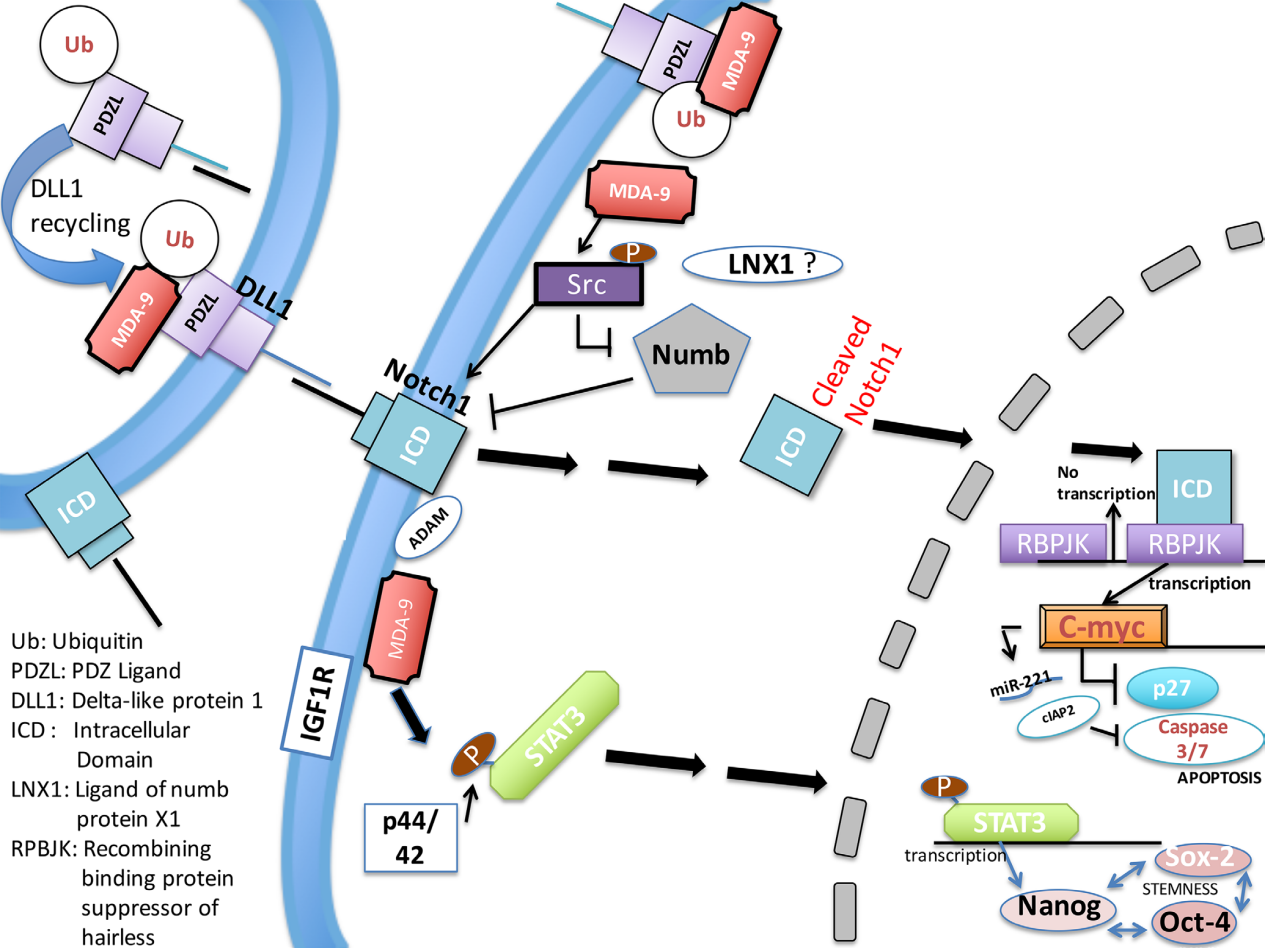
Schematic representation of MDA-9-mediated regulation of GSC survival and stemness MDA-9 regulates stem cell survival and pluripotency by regulating several molecular activities and promoting defined gene expression changes. The survival pathway is affected by the expression, degradation or activation of the constituents of the NOTCH1/C-Myc signaling pathway. Stemness is regulated by the STAT3/Nanog signaling pathway, which is likely regulated by p-p44/42 and IGF-R1. Ub = Ubiquitin, PDL = PDZ ligand, ICD = Intracellular domain, DLL1 = Delta-like protein 1, LNX1 = Ligand of numb protein 1, RBPJK = Recombining binding protein suppressor of hairless.

GSCs, also called glioma initiating cells, are considered defining elements in the carcinogenic process, hypothesized to represent critical constituents of invasion, angiogenesis, cancer cell resistance to therapy and escape of tumor cells from dormancy (tumor recurrence /relapse occurring after an initial therapeutic response) [36–38]. MDA-9 is a diagnostic marker of tumor aggression and grade, and a positive association has been reported between MDA-9 expression and glioma stage [23]. We now demonstrate a fundamental and central role of MDA-9/Syntenin (SDCBP) as an upstream regulator of stemness and GSC survival in GBM. MDA-9 contributes to GSC cell-cell/cell-matrix adhesion, angiogenesis and invasion. Stem cell-mediated cancer progression is a major clinical problem [6–9, 11, 18, 20] and is accentuated as a significant contributor to therapy-resistance and cancer relapse [4]. *mda-9* expression positively correlated with stemness as confirmed by a direct association between expression of *mda-9* and stem cell markers and genes, in both GBM patient samples and cell lines. Loss or gain of *mda-9* expression led to a corresponding loss or gain of cell surface stem markers, respectively (Figure 2A, 2B, 2C, and Table S2), as well as changes in recognized self-renewal/pluripotency genes including *Nanog, Oct4, Sox2* and *c-myc* (Figure 1A, 1B; Figure S1, S2; Table 2). *mda-9* expression was also significantly higher in GSCs than NSGCs (Table 1) and both were dramatically elevated as compared to corresponding normal astrocyte stem cells (Figure 2A). *mda-9* also regulated STAT3 expression (Figure 3, Figure S2), which is a key contributor to cellular transformation and tumor maintenance in many cancer contexts, including GBM [16]. Activation of a STAT3-mediated transcriptional network correlates with mesenchymal GBM transformation and poor prognosis (30, 31, 39). STAT3 also regulates self-renewal in cancer stem cells by systematically regulating canonical stemness genes including *Nanog, Sox2, Oct4* [16, 29, 30] and *myc* [40]. NANOG also acts as a master switch of the central stemness transcriptional network, as OCT4/SOX2 bind to the proximal region of the Nanog promoter stimulating *Nanog* expression [15]. NANOG, SOX2 and OCT4, also bind to their individual promoter's, thus sustaining a unified auto-regulatory network of cell pluripotency and self-renewal [15]. Our data reveal that *mda-9* is a key regulator of this core GSC regulatory system through regulation of STAT3 (Figure 9).

STAT3 can be regulated by SRC, IGF-1R, and p-44/42 [29–33]. Phosphorylated p-44/42 (T202/Y204) and SRC (T417, Y418) phosphorylate STAT3 at position Y705. Our data indicates that MDA-9 regulates STAT3 by controlling IGF-1R (Figure 4B), p-44/42 (Figure 4A) and Src (Figure 6B) signaling. MDA-9 also regulates the activity of FAK [23, 24], RAF and RKIP [25] and it ultimately controls the activation of p-44/42. MDA-9 physically interacts with c-SRC through its PDZ binding motifs and is essential for activation of SRC [23, 41]. These data demonstrate that MDA-9 influences GSC stemness on multiple molecular levels. The higher expression of MDA-9 in GSCs than in normal astrocyte stem cells may indicate that GSCs are more dependent on *mda-9* expression than their corresponding non-cancer stem cells. The potential “addiction” of GSCs to MDA-9 is an area of current investigation.

Another critical pathway in stem cell biology is the NOTCH pathway [16]. NOTCH signaling plays an important role in development by regulating cell-fate determination, cell survival, and proliferation [17]. Activation of NOTCH receptors occurs through binding with a number of distinct ligands (including delta-like 1 / DLL1, jagged 1). Upon ligand binding, the intracellular NOTCH domain (ICD) is cleaved and translocates into the nucleus, where it regulates downstream target gene transcription. Aberrant NOTCH signaling promotes tumorigenesis [17]. Recently, a role of the NOTCH signaling pathway in promoting self-renewal of both normal and cancer stem cells has been demonstrated [17, 42]. Our data indicates that MDA-9 regulated NOTCH1 activity on two levels. MDA-9 controlled the expression of NOTCH1 in cells by degradation (Figure 6B), through NUMB, a NOTCH binding ubiquitin ligase [13, 43]. In the presence of p-SRC, NUMB is phosphorylated and then degraded, preventing it from degrading NOTCH1 [44]. In the absence of MDA-9, SRC is not activated to p-SRC, and this leads to higher expression of NUMB resulting in degradation and a decrease in the levels of total NOTCH1.

MDA-9 also controls NOTCH1 activity by regulating NOTCH1 activation through expression of DLL1, the ligand of the NOTCH1 receptor (Figure 6A). The intracellular PDZ binding motif of DLL1 regulates DLL1 protein stability [45], DLL1 trafficking and signaling activity. DLL1 ubiquitination is not required for its internalization, but is necessary for its recycling back to the plasma membrane and efficient interaction with NOTCH1 [46]. MDA-9 can regulate the expression of DLL1 on the cell surface by regulating the interaction between DLL1 and ubiquitin. An effect of MDA-9 on DLL1 has been reported in zebrafish stem cells [47]. The c-terminal of MDA-9 binds to ubiquitin [48], and its PDZ domain may then bind to the PDZ binding motif of DLL1, and this interaction regulates the expression of DLL1 on the surface of GSCs. In the absence of MDA-9 this interaction is altered, leading to decreased DLL1 surface expression. This further reduces the interaction of NOTCH1 with its ligand DLL1, leading to decreased activation of NOTCH1, reduced translocation of the intracellular domain (ICD) of Notch1 to the nucleus and decreased transcription of NOTCH1 target genes.

NOTCH1 directly regulates C-Myc expression [49]. The ICD of NOTCH1 translocates to the nucleus and binds to the promoter of the transcription factor RBPJK, which regulates *c-myc* expression [50]. The binding of NOTCH1 to the promoter region of RBPJK promotes expression of RBPJK, leading to expression of *c-myc*. In MDA-9 kd cells the ICD of NOCTH1 is unable to translocate to the nucleus, preventing transcription of RBPJK (Figure 7A), thereby inhibiting elevated *c-myc* expression (Figure 7B).

Elevated MYC proteins are associated with many cancers and correlate with cancer risk and poor patient survival [19, 51]. Activation of MYC is linked to cellular growth, proliferation and metabolism. C-Myc controls the balance between stem cell self-renewal and differentiation in normal cells. In GBM, C-Myc is essential for GSC regulation and maintenance [34, 35]. C-myc also controls the proliferation of cells by regulating cell cycle modulators including the cyclin-dependent kinase inhibitor, p27, which is a critical target of C-Myc [52]. SRC has also been shown to negatively regulate p27 and elevated levels of p27 cause arrest of tumor growth and apoptosis [53]. Additionally, p27 can suppress SOX-2 [54], which leads to apoptosis in stem cells [55]. Our data revealed that kd of *mda-9* decreased SRC, *Sox-2* and C-Myc activities, whereas p27/kip-1 expression was increased, culminating in apoptosis of GSCs (Figures 3B, 5A, 6B, 7B). Another anti-apoptotic molecule cIAP2, was also regulated by MDA-9 in GSCs (Figure S2). IAP family members, XIAP, cIAP1, cIAP2, NAIP and survivin, are expressed at higher levels in CD133 positive than in CD133 negative GBM [56], and these anti-apoptotic proteins contribute to GSC survival under adverse conditions. Kd of *mda-9* expression decreased expression of cIAP2 (Figure S2), which also participated in induction of apoptosis (Figure 5A).

The current data suggests that MDA-9/syntenin (SDCBP) is part of a complex, tightly regulated connectivity network that confers self-renewal, survival and tumor progressive properties to GSCs [57]. Stemness, initially defined by the expression of cell surface markers and stem cell genes, is a property shared by normal stem cells and GSCs [58]. MDA-9 appears to regulate stemness through similar pathways in both normal astrocyte stem cells and GSCs. However, GSCs appear to be more dependent on (“addicted” to) MDA-9, with significantly elevated expression (Figure 2A), for maintenance and survival than normal stem cells. Forced elevated expression of MDA-9 in normal astrocytes increased their invasiveness, self-renewal and the overall proportion of stem cells, but it did not render these cells tumorigenic. The regulation of stemness by MDA-9 is not exclusive to GSCs, but elevated expression enhances GSC survival, invasion, angiogenesis, and self-renewal. MDA-9 is capable of regulating multiple aspects of stem cell phenotypes simultaneously, validating a critical role in determining GSC stemness. *mda-9* can regulate the central transcriptional network of stem regulating genes, additional pluripotency genes, and affects interrelated pathways crucial for stem cell survival (Figure 9). Considering the pivotal role of MDA-9 in determining both GSC aggressiveness and survival, directly targeting MDA-9 expression or its interaction with effector interacting proteins using genetic or pharmacological approaches may provide a unique opportunity to develop targeted therapies for this important component of cancer pathogenesis.

## MATERIALS AND METHODS

### Cell lines and tissue samples

Specimens of human primary normal and malignant brain tumors (*n* = 50) were collected from subjects who underwent surgical removal of their brain tumors. All subjects were informed of the nature and requirements of the study and provided written consent to donate their tissues for research purposes. Informed consent was obtained according to Origene and the research proposals approved by the Institutional Review Board at the VCU TDAAC. The human glioma cell line U-1242-luc-GFP was kindly provided by Dr. Kristofer Valerie (VCU). U-1242/luc-GFP (U-1242-Luc), cells were cultured in DMEM medium supplemented with 10% fetal bovine serum and antibiotics. Isolated non-stem glioma cells (NSGCs), based on lack of expression of CD133 and CD44, were cultured similarly in monolayer culture. Normal human astrocytes (HA) were obtained from Clonetics, USA and grown in Astrocyte Basal Media (ScienCell, CA) supplemented with Astrocyte growth supplement (ScienCell, CA). The cumulative culture length of the cells was less than 6 months after resuscitation. Early passage cells were used for all experiments and authenticated. All the cell lines were frequently tested for mycoplasma contamination using a mycoplasma detection kit from Sigma. All primary GBM cells were authenticated by IDEXX Bioresearch (Columbia, MO).

### Isolation and culture of human GBM, putative GSCs and NSGCs

Human GBM GSCs and NSGCs were isolated from GBM tissue from surgical specimens and from established U-1242/luc-GFP GBM cells. GBM tissue samples were dissociated and digested samples were filtered with 70 μm nylon cell strainer (BD) and resuspended in stem cell medium comprised of DMEM/F-12 50:50 containing K27 supplements, glutamine 2 μmol/L (Invitrogen), basic fibroblast and epidermal growth factors (PeproTech, 20 ng/mL each) for continuous culturing [59]. All primary cells were cultured as suspended spheres in ultra-low attachment T25 or T75 culture disks (Corning) in Essential 8 medium (Invitrogen) and analyzed prior to 5 passages to form GSC enriched neurospheres. Neurospheres were dissociated and then labeled with CD44 and CD133 antibody (Miltenyi Biotech) to isolate both pro-neural and mesenchymal GSC subtypes. Stained cells were sorted through a BD Aria II sorting station. Antibody negative and positive cell populations were counted and collected for further culturing. GSC and NSGC populations were counted and collected for further culturing. Xenografted human GSCs were isolated from mice and analyzed for cell surface and intracellular proteins by FACS. The GSCs were cultured in ultra-low attachment plates and flasks with Essential 8 medium (Invitrogen), unless indicated. Isolated NSGCs were cultured in monolayer with complete DMEM medium.

### Isolation and culture of primary human astrocyte stem cells

Primary normal human astrocytes were cultured in ultra-low attachment plates and flasks (Corning) in Astrocyte media (ScienCell, CA). The cells were stained with CD44 and CD133 antibodies, sorted and cultured further under ultra-low attachment conditions.

### Self-renewal assay

Sphere-forming assays were used to determine clonogenic growth potential *in vitro* of both normal and neoplastic cells [60]. Sorted GSCs and NSGC populations were diluted to a density of 500 cells/ml. 2 μl of the diluted cell suspension was plated per well in a 96 well ultra low attachment plate (Corning Inc., Corning, NY, USA), and 150 μl of serum-free medium was added, cultures were then observed daily (*n* = 96). Additionally, flow cytometry with CD44 and CD133 antibody (Miltenyi Biotech) was performed to assess the stem populations.

### Gene expression arrays, and analyses

TissueScan Brain Cancer Tissue cDNA array I, containing 46 malignant (covering four stages) and 2 tumor-adjacent normal tissue cDNAs, were obtained from Origene Technologies, (Rockville, MD, USA). This array was analyzed for *mda-9, c-myc, Oct4* and *Sox2* expression using taqman probes (Invitrogen) according to the manufacturer's protocol. A human GSC array (Qiagen) was used according to the manufacturer's protocol to analyze a clinical GBM sample VG2 (sh*con*) and an *mda-9* kd clone of VG2 (sh*mda-9*). Eighty-four genes were studied. The data was analyzed using the Qiagen web-based PCR array data analysis software.

### Promoter reporter assays

Luciferase reporter assays were performed using 2×10^5^ cells infected with either Ad.5/3.sh*con* or Ad.5/3.sh*mda-9*. Twenty-four hours post-infection, cells were transfected with an RBPJK-responsive luciferase reporter construct with Lipofectamine 2000 as described [23]. Cell lysates were harvested and luciferase activity was measured using a Dual-Luciferase Reporter Assay system (Promega) according to the manufacturer's instructions. Luciferase activity was normalized to Renilla activity, and data represent the average of triplicates ± S.D.

### Reverse transcription polymerase chain reaction

Total RNA was isolated by TRIzol extraction (Invitrogen) and purified using the RNeasy kit (Qiagen). First-strand cDNA was synthesized with SuperScript III reverse transcriptase (Invitrogen). Quantitative PCR studies were carried out by using the TaqMan Gene expression assays (Invitrogen), and were normalized to 18S expression (Invitrogen). Probe details are provided in supplemental information.

### Western blotting

Cells were lysed on ice in lysis buffer (20 mM Tris-HCl (pH 7.5), 150 mM NaCl, 1 mM Na_2_EDTA, 1 mM EGTA, 1% Triton-100, 2.5 mM Sodium pyrophosphate, 1 mM β-glycerophosphate, 1 mM Na_3_VO_4_, 1 μg/ml Leupeptin). Protein samples were prepared after protein concentration was determined, and were loaded onto 8% SDS-PAGE for immunoblotting detection. For densitometry evaluation, X-ray films were scanned and analyzed with ImageJ software (National Institutes of Health [NIH]). Antibody details are provided in supplemental information.

### Flow cytometry sorting and analysis

CD44, CD133, NOTCH1, DLL1, staining and Annexin V staining were performed according to the manufacturer's instructions, followed by flow cytometry analysis using BD DIVA.

### Intracellular flow cytometry

STAT3, p-STAT3, p44/42, p-p44/42, p-Src, and Numb proteins were assessed by intra-cellular flow cytometry [61, 62]. Cell fixation, permeabilization and antibody staining were performed according to the manufacturer's instructions, followed by flow cytometry analysis using BD DIVA.

### Tumorigenicity studies

All experiments and procedures involving mice were approved by the Institutional Animal Care and Use Committee of Virginia Commonwealth University. For the intracranial brain tumor model, athymic female NCr-nu/nu mice (National Cancer Institute-Frederick) were used (*n* = 10 per group). Mice were anesthetized through i.p. administration of ketamine (40 mg/kg) and xylazine (3 mg/kg) and immobilized in a stereotactic frame (Stoelting). Intracerebral injections of 1.5 × 10^4^ cells in 2 μL per mouse were done using an automated injector (Stoelting) as described earlier [23, 59]. Tumor burden was determined by bioluminescent imaging [63]. Animals of each group were monitored until they reached the point of euthanization according to the VCU-IACUC approved protocol and survival data was analyzed.

### Peptide blocking/activation studies

1 × 10^5^ control and treated GSCs were cultured in 6-well ultra-low attachment plates. NOTCH1 blocking peptide (Biovision) and DLL1 peptide (Abcam) were used at a concentration of 10 μg/ml and incubated with cells for 48 hours. After incubation the cells were stained and analyzed for viability, spheroid size and structure.

### Live/dead cell assay

Live/Dead cell staining was performed according to the manufacturer's instructions (Invitrogen), followed by imaging by laser confocal microscopy (Leica). The images were analyzed by Zen software.

### Histology

Mice were euthanized according to the veterinarian's suggestions (approximately 1-6 months from intracranial injection). The mice were carefully dissected to obtain the brain tissue. Paraffin-embedded tissues were sectioned at 4-μm thickness and stained with Haematoxylin and Eosin.

### shRNA knockdown

shRNA sequences were obtained through Qiagen with the following sequences: 5′-TTGACTCTTAAGATTATGTAA-3′ (sh*mda-9* #3) and 5′-TGGGATGGTCTTAGAATATTT-3′ (sh*mda-9* #4). Ad.5/3.sh*mda-9* was constructed as previously described [23] using the following primer sequences: forward:5′-GCCTGCTTTTATCTTTGAACATATTATTAAGCGAATGAAGCCTAGTATAAGAAAAGCCTAATGGACCACACCATTCCTGAG-3′ and reverse: 3′-CGGACGAAAATAGAAAC TTGTATAATAATTCGCTTACT TCGGATCATATTACTTTTCGGATTACCTGGTGTGGT AAGGACTC-5′.

### Statistical analysis

For all *in vitro* and *ex vivo* experiments, statistical analyses were conducted using Student's t test and ANOVA (Microsoft Excel). For *in vivo* studies, statistical analyses were performed using the Kaplan-Meier method (survival studies). Pearson's correlation coefficient (R) and coefficient of determination (R^2^) were calculated for correlation analysis. The data from clinical samples were analyzed using Microsoft Excel's multiple regression analysis tool. All statistical tests were two-sided, and p values ≤ 0.05 and ≤ 0.01 were considered to be significant and highly significant, respectively. Confidence interval (CI) of 95% was considered statistically significant. Patient data was analyzed using correlation heatmap and cluster analysis tools (Plotly Technologies Inc. Montreal, QC). The RT^2^ Profiler PCR Array Data Analysis software was used to study the statistical significance of cancer stem cell array data, and a minimum of an ~4-fold decrease was analyzed by selecting the statistical boundary for Log_10_shmda-9 del del CT/ Log_10_shcon del del CT as 4.

## SUPPLEMENTARY MATERIALS


