# Supplementary material for: Novel function of MDA-9/Syntenin (SDCBP) as a regulator of survival and stemness in glioma stem cells
Source: Oncotarget. 2016 Jul 26;7(34):54102–19. doi: 10.18632/oncotarget.10851 (PMC5342330; doi:10.18632/oncotarget.10851)
Supplement: Supplementary file 1 [file oncotarget-07-54102-s001.pdf]

Novel function of MDA-9/Syntenin (SDCBP) as a regulator of survival and stemness in glioma stem cells

Supplementary Material

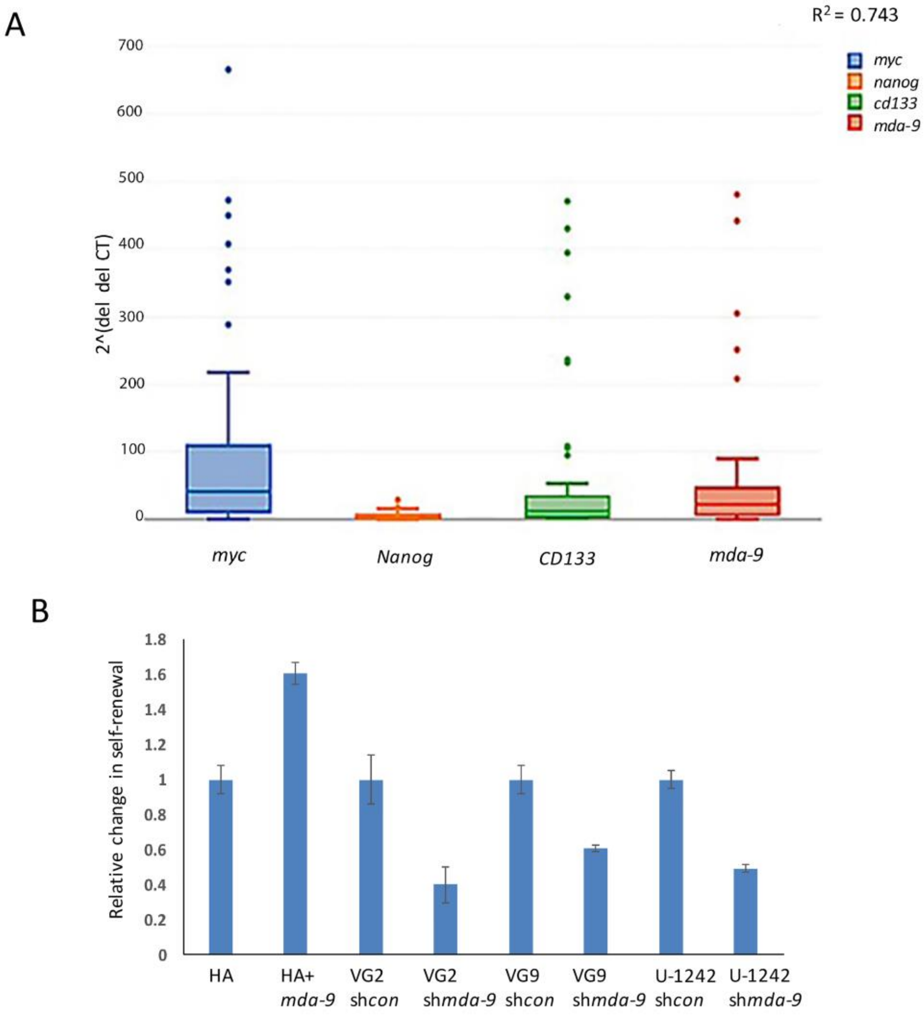

Figure S1: *mda-9* expression is strongly correlated with expression of stemness associated molecules and regulate self renewal of stem cells. (A) Graphical plot showing the expression and association of *c-myc*, *Nanog*, *CD133* and *mda-9* in different clinical samples (n = 48). (B) Self-renewal analysis in primary human astrocytes (HA), and *mda-9* overexpressing HA stem cells, as well as in control and *mda-9* knockdown stem cells from VG2, VG9 and U-1242 GBM cells.

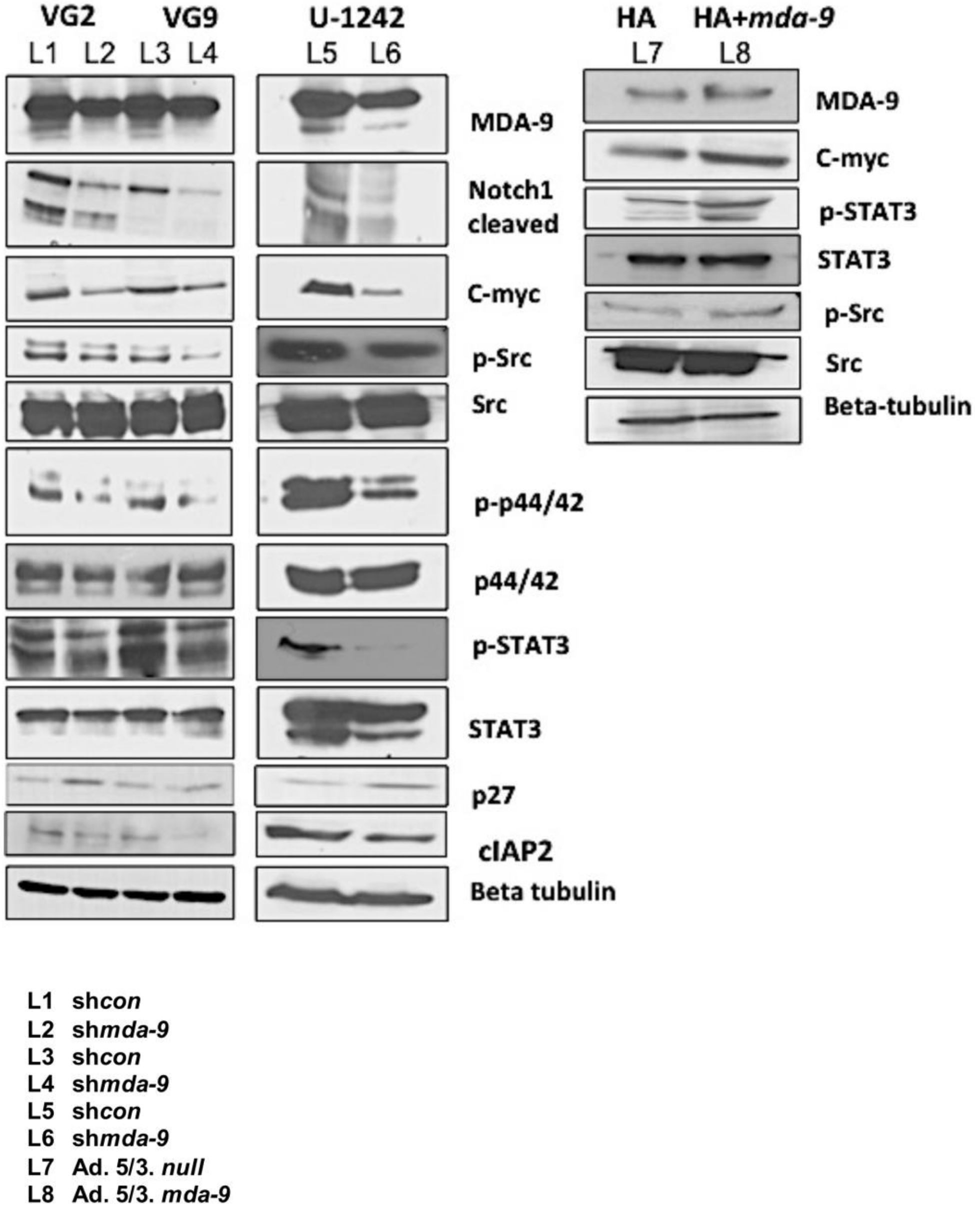

Figure S2: *mda-9* regulates molecules and pathways associated with self-renewal and survival. Left panels, expression of the indicated proteins by Western blot analysis in control and *mda-9* knockdown stem cells from VG2, VG9 and U-1242 GBM cells. Right panel, expression of the indicated proteins by Western blot analysis in stem cells from control and *mda-9* overexpressing HA.

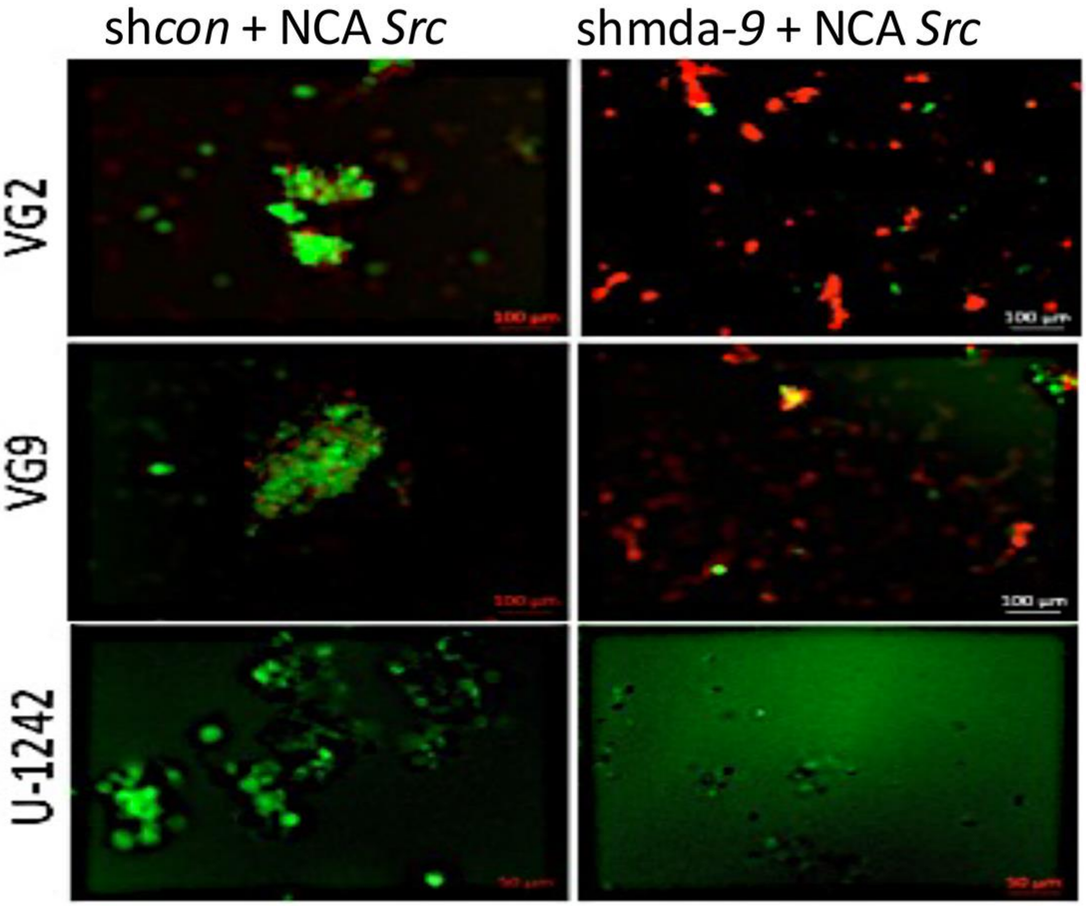

Figure S3: ***mda-9*** **regulates stem cell phenotypes through Src activation.** Live image analysis of sh*con* and *mda-9* kd VG2, VG9 and U-1242 GSCs overexpressing non constitutively activated (NCA) *Src*. Scale bars, 100 μm.

Methods

Antibodies

MDA-9: Abnova (H00006386-M01) (western blot); C-MYC: Abcam (ab32072) (western blot); STAT-3: (Flow cytometry) (western blot); P-STAT3: CST ((Y705) (M9C6) #9145 (Flow cytometry) (western blot); p44/42: (Flow cytometry) (western blot); P-p44/42: (Flow cytometry) (western blot); NOTCH1-PE: BD Pharmingen (MHN1-519) (Flow cytometry); DLL1-APC: Miltenyi Biotec (clone: MHD1-314) (Flow cytometry); Numb: Abcam (ab123891) (Flow cytometry); Src: CST #2108 (western blot); p-Src: BD (560094) (Flow cytometry) CST #6943 (western blot); CD44-PE: BD Pharmingen (550989) (Flow cytometry); CD24-FITC: BD Pharmingen (555427) (Flow cytometry); CD133-APC: Miltenyi Biotec (130-090-826) (Flow cytometry); SOX2: CST (#3579) (immunofluorescence); OCT4: CST (#2840) (immunofluorescence).

Probes

mda-9	Hs01045460_g1
myc	Hs00153408_m1
Nanog	Hs04399610_g1
Sox2	Hs00415716_m1
Oct4	Hs04260367_gH
CD133	Hs01009250_m1
Notch1	Hs01062014_m1
18S	Hs99999901_s1

Overexpression studies

The genomic sequence of *mda-9/syntenin* was amplified by PCR using genomic DNA as template and primers, sense: 5'-CTGCAAAAATGTCTCTCTATCC-3′ and anti-sense: 5'-GGTGCCGTGAATTTTAAACCTCAG-3'. The PCR product was cloned into a pREP4 expression vector and then it was digested and released with Xho and BamH1 and subcloned into the pcDNA3.1 (+hygro) plasmid (Invitrogen).

The DNA fragment (990-bp) having the *mda-9/syntenin* gene was isolated from plasmid p0tg-CMV–MDA-9 [1] and cloned between BglII and EcoRV sites downstream of the cytomegalovirus (CMV) promoter in the plasmid pSh-CMV. The shuttle plasmids were recombined with genomic DNA of Ad.5/3.*Luc1* vector as previously described [1] to derive plasmids pAd.5/3.sh*mda-9* or pAd.5/3.*mda-9*. The resultant plasmids were digested with PacI to release the recombinant adenovirus genomes and then transfected into human embryonic kidney (HEK)–293 cells to rescue the corresponding Ad.5/3-based vectors. The rescued viruses were amplified using HEK-293 cells and purified by cesium chloride double ultracentrifugation using standard protocol, and the titers of infectious viral particles were determined by plaque assay using HEK-293 cells as described [1]. The cells were infected with Ad. 5/3.sh*con* and Ad.5/3.sh*mda-9* (1000 v.p./cell) in serum free media for 4 hours and the media was replaced with fresh complete media.

pcDNA3-cmyc plasmid (Addgene #16011), pCMV5 human p27/kip-1 (Addgene #14049), EF.STAT3C.Ubc.GFP (Addgene #24983) were used for forced expression of *c-myc*, *p27/kip-1* and *CA-Stat-3* respectively. The CA-Src and Src plasmid were kind gifts from Dr. Jeffrey N. Bruce. The plasmids were transfected into CSCs by incubating with Fugene 6

according to the manufacturer's instructions, under overnight shaking, and then replaced with fresh complete media.

Supplementary Reference

1. Kegelman TP, Das SK, Hu B, Bacolod MD, Fuller CE, Menezes ME, Emdad L, Dasgupta S, Baldwin AS, Bruce JN, Dent P, Pellecchia M, Sarkar D, et al. MDA-9/syntenin is a key regulator of glioma pathogenesis. Neuro Oncol. 2014;16:50-61.
